# Convergent evolution in social swallows (Aves: Hirundinidae)

**DOI:** 10.1002/ece3.2641

**Published:** 2016-12-20

**Authors:** Allison E. Johnson, Jonathan S. Mitchell, Mary Bomberger Brown

**Affiliations:** ^1^ Department of Ecology and EvolutionUniversity of ChicagoChicagoILUSA; ^2^ Department of Ecology and Evolutionary BiologyUniversity of MichiganAnn ArborMIUSA; ^3^ School of Natural ResourcesUniversity of NebraskaLincolnNEUSA

**Keywords:** coloniality, convergent evolution, morphology, sociality

## Abstract

Behavioral shifts can initiate morphological evolution by pushing lineages into new adaptive zones. This has primarily been examined in ecological behaviors, such as foraging, but social behaviors may also alter morphology. Swallows and martins (Hirundinidae) are aerial insectivores that exhibit a range of social behaviors, from solitary to colonial breeding and foraging. Using a well‐resolved phylogenetic tree, a database of social behaviors, and morphological measurements, we ask how shifts from solitary to social breeding and foraging have affected morphological evolution in the Hirundinidae. Using a threshold model of discrete state evolution, we find that shifts in both breeding and foraging social behavior are common across the phylogeny of swallows. Solitary swallows have highly variable morphology, while social swallows show much less absolute variance in all morphological traits. Metrics of convergence based on both the trajectory of social lineages through morphospace and the overall morphological distance between social species scaled by their phylogenetic distance indicate strong convergence in social swallows, especially socially foraging swallows. Smaller physical traits generally observed in social species suggest that social species benefit from a distinctive flight style, likely increasing maneuverability and foraging success and reducing in‐flight collisions within large flocks. These results highlight the importance of sociality in species evolution, a link that had previously been examined only in eusocial insects and primates.

## 1 Introduction

Animal morphology and behavior are inextricably linked, with particular morphologies permitting particular behaviors, and behavioral innovation producing novel selective pressures on relevant morphologies. For example, the resonant vocalizations of sandhill cranes (*Antigone canadensis*) require the extension of the trachea into the sternum (Johnsgard, 1983), and the territorial displays of red‐winged blackbirds (*Agelaius phoeniceus*) are less effective against intruders without the males’ bright red wing epaulets (Yasukawa & Searcy, 1995). Changes in behavior have long been implicated in initiating changes in morphological traits by affecting how species interact with their environment and by altering selective pressures (Duckworth, 2008; Lapiedra, Sol, Carranza, & Beaulieu, 2013). A number of studies have examined how behaviors associated with ecological differences between species, such as preference for certain habitats, direct morphological evolution (e.g., Desrochers, 2010; Douglas & Matthews, 1992; Losos, 1990; Miles & Ricklefs, 1984; Streelman, Alfaro, Westneat, Bellwood, & Karl, 2002).

Social behavior should play a similar role in influencing morphological evolution, with species changing in accordance with the new physical demands involved in performing social or group behaviors, but social behavior's influence has been rarely studied in nonextinct species. For instance, ecological influences such as the cluttered foraging habitat of bats have been shown to influence wing morphology (Kalcounis & Brigham, 1995; Saunders & Barclay, 1992), but a similar pressure from social behavior to prevent collisions in large social roosts could produce repeated convergence of wing morphology. Social behavior has been linked to the evolution of morphology in eusocial insects, with diversity in number of castes and caste morphology linked to colony size and complexity (Oster & Wilson, 1978; Bourke, 1999; Fjerdingstad & Crozier, 2006). In mammals, the relationship between brain morphology and social behavior has been well studied (e.g., Dunbar, 1995; Noonan et al., 2014; Shultz & Dunbar, 2007, 2010), but little work has been carried out to link sociality to morphological evolution more broadly in vertebrates.

**Figure 1:**
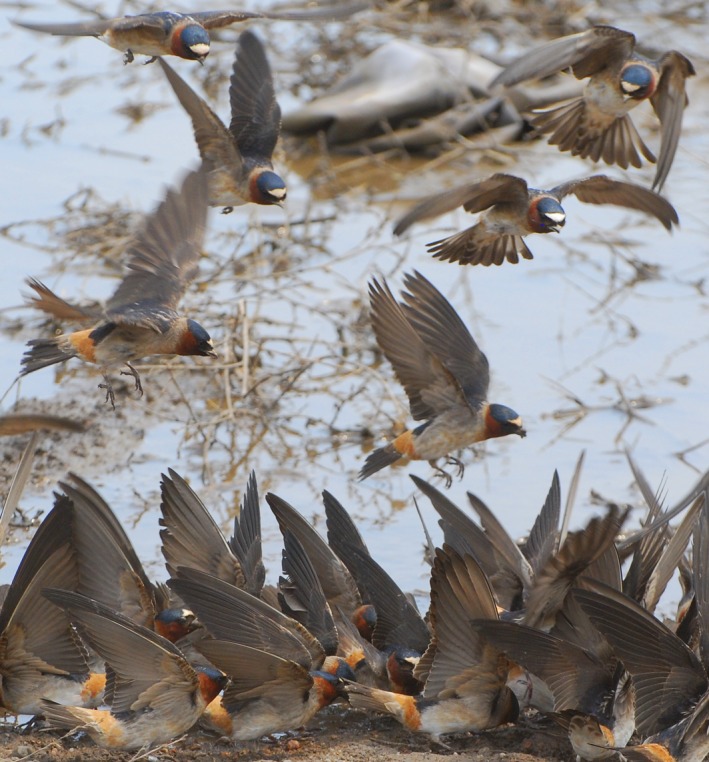
A flock of cliff swallows (*Petrochelidon pyrrhonota*), one member of the Hirundinidae family, collecting mud for nest building. Photograph taken by Joel G. Jorgensen

To better understand the role of social behavior in influencing morphological evolution, we compared the evolution of morphological features important to flight and foraging to the evolution of social behaviors in the socially diverse bird clade, the Hirundinidae (swallows and martins, see Figure 1 for an image of one member of the Hirundinidae family). The Hirundinidae consist of 84 species distributed worldwide, which have a long history of field studies focused on social behaviors, foraging strategies, and general natural history (Beecher, Beecher, & Lumpkin, 1981; Møller, 1987; Brown, 1988; Brown & Brown, 1996, 1998, 2000, 2011, 2013, 2014, 2015; Turner & Rose, 1989; Turner, 2004; Sheldon, Whittingham, Moyle, Slikas, & Winkler, 2005; Roche, Brown, & Brown, 2011; Brown, Brown, & Roche, 2013; Brown et al., 2015, 2016). All species are obligate aerial insectivores (Turner, 2004; Turner & Rose, 1989), a foraging strategy that requires agile, acrobatic flight. However, they exhibit great diversity in their degree of sociality (e.g., solitary to colonial breeding, solitary to group foraging). Breeding group sizes can range from a single pair to as many as 6,000 pairs (Brown et al., 2013; Turner, 2004; Turner & Rose, 1989). Foraging group sizes range from individuals and pairs foraging in isolation to flocks of hundreds of individuals foraging in close proximity (Brown & Brown, 1996; Graves, 2013; Ricklefs, 1971; Santema, Griffith, Langmore, Komdeur, & Magrath, 2009). Group foragers most often exploit swarming or aggregating species of insects, including mass emergences, mating swarms, insects caught in local convection currents or sheltering in the lee side of hills under inclement conditions (Brown & Brown, 1996). Insects utilized by group foragers are typically smaller than those consumed by nonsocial foragers (Brown & Brown, 1996; Bryant & Turner, 1982; Quinney & Ankney, 1985; Turner, 1982).

**Figure 2:**
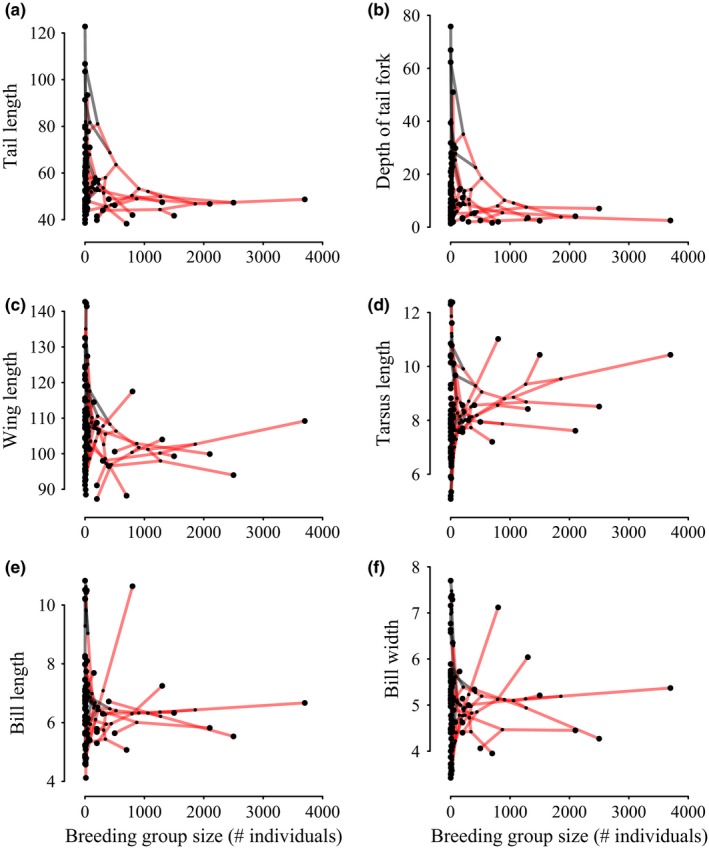
Phylomorphospaces of morphological trait values compared to maximum observed breeding group size for (a) outer tail length, (b) depth of tail fork, (c) wing length, (d) tarsus length, (e) bill length, and (f) bill width. All morphological values are scaled by tarsus except tarsus length

Using published behavioral and ecological data from 40 sources (see Table A2 in Appendix S1), measurements of 525 museum specimens, and a phylogeny from Sheldon et al. (2005) encompassing 75 of the 84 swallow species, we asked how breeding and foraging social behaviors are correlated with the evolution of external morphology. We define sociality as intraspecific interactions that occur during breeding and foraging. In examining the morphological and social data, a pattern of reduced morphological diversity in social species is apparent, with solitary species showing a wider range of variation across all measured traits (see Figure 2). This pattern has four potential explanations: (1) It could be a spurious result of a small number of social species; (2) it could be a spurious result from a single ancestral swallow that became social, and all subsequent descendants inherited similar morphology (phylogenetic autocorrelation); (3) social species could have an additional constraint, such as occurring only in a specific habitat, that selects for a particular morphology; (4) social habits may exert direct selection on morphology by increasing competition between individuals in a social group for the same resources (including flight space or aerodynamic requirements for maneuverability) promoting morphological convergence.

In this study, we explore these different explanations and attempt to determine which is most likely to explain the observed patterns. We use a liability threshold model to understand the pattern of social evolution along the swallow phylogeny (Felsenstein, 2012; Revell, 2014). Models of discrete character evolution that rely on a transition matrix assume a consistent rate of evolution across the whole tree, making similarly sized clades with different levels of heterogeneity problematic. After reconstructing the evolution of social behavior, we used various metrics of convergence (Arbuckle, Bennett, & Speed, 2014; Stayton, 2015) to test whether the external morphology of social species converged on each other and quantified the strength of that convergence.

## 2 Materials and Methods

### 2.1 Morphological measurements

We measured six external morphological traits on 525 museum specimens (skins) from 73 species of swallows and martins (data deposited on Dryad and available in Table A1 in Appendix S1). These species represent 19 of the 21 genera in the Hirundinidae, excluding only *Haplochelidon* and *Alopochelidon*, both of which contain only one species (Clements et al., 2014; Dickinson, 2003). To balance time spent measuring a single species against sample size, we measured five males and five females of each species whenever possible. For species without five males and females in the museum collections, we measured all available specimens. For seven species, we were only able to measure one specimen (see Dryad data file). To account for how specimens shrink over time, are prepared using different techniques, and the fact that plumage can vary by season, we measured specimens that were of approximately the same age, collected at the same time of year, and made by the same preparator, when possible. Specimens used in our analyses are housed in the collections of the Field Museum of Natural History (Chicago, IL), the Smithsonian Institution (Washington, DC), and the Louisiana State Museum of Natural Science (Baton Rouge, LA).

The following traits were measured for all specimens: wing length, depth of the tail fork, outer tail feather length, tarsus length, bill length, and bill width. For all specimens, the length of each unflattened, closed wing (from the anterior most part of the wrist joint to the tip of the outermost primary) was measured to the nearest 1 mm with a stoppered wing ruler; the length of the middle tail feather and the two outermost tail feathers (from the emergence from the skin to the distal most point) were measured to the nearest 1 mm with a ruler; the length of each tarsus (from the proximate end of the tarsometatarsus to the hallux) was measured to the nearest 0.1 mm with calipers; and the length and width of the exposed bill (length from the proximate end of the exposed bill to the tip along the ridge of the upper mandible and width of the exposed mandibles at the level of the nostrils) were measured to the nearest 0.1 mm with calipers. While many studies examining wing morphology include Kipp's distance (distance between longest primary feather and the first secondary feather when the wing is closed; Kipp, 1942; Dawideit, Phillimore, Laube, Leisler, & Böhning‐Gaese, 2009), we were unable to take this measurement because of the condition or preparation of the specimens used. The outermost primary feather length has been used in other studies of morphology and wing evolution in swallows and serves as a practical proxy (Brown & Brown, 1996, 2011, 2013; Price, Brown, & Brown, 2000). One person took all morphometric measurements (MBB), and thus, no corrections to the data for multiple measurers were necessary. Repeatability estimates for these same body size measurements (cliff swallow, *Petrochelidon pyrrhonota*, Figure 1), made by MBB, were all statistically significant (*p *<* *.001; see Brown & Brown, 1998). Measurements were taken on both left and right sides of each specimen (when appropriate) and averaged. We evaluated the tail shape reflected in the depth of the tail fork by subtracting the middle tail length from the mean outer tail length. All categories (wing, outer tail, depth of tail fork, tarsus, bill length, and bill width) were averaged across all individuals (male and female) measured for each species. For all analyses described below, trait values are all relative to the length of the tarsus to control for variation in body size.

### 2.2 Behavioral scoring

We used two measures of sociality—breeding behavior and foraging behavior. We chose two metrics as these forms of sociality may result in differing selective pressures and while most socially breeding species forage socially, some solitary breeding species also forage socially. For breeding behavior, we performed a primary literature search to find the maximum reported breeding group size for every species with sufficient behavioral data recorded (see Table A2 and associated references in Appendix S1 for all citations). All species with appropriate data were then categorized as either social or solitary. Social species are those species that have been documented nesting in groups of five or more pairs. The two species documented as forming “colonies” of two to five pairs (*Progne sinaloae* and *Notiochelidon murina*) utilize existing cavities rather than constructing them and are only found in groups larger than pairs when cavities are spaced near one another. They do not appear to exhibit any social cohesion, and we therefore classified these species as solitary.

Foraging behavior was determined based on a primary literature search. Foraging behavior was divided into two categories, pairs and groups. The pair foraging category represents the solitary category for foraging behavior and was defined as species that have been observed primarily to forage solitarily or as breeding pairs only; most solitary species will forage with their mate over the course of the breeding season (Turner, 2004). The group foraging category was defined as species observed to forage in groups beyond the breeding pair. Some species were placed in the pairs or group categories based on descriptions of behavior if specific foraging group size counts were lacking. One species, *Notiochelidon flavipes,* had data on foraging behavior and was included in the foraging data set, but lacked data on breeding behavior.

As engaging in one social behavior may relate to the propensity to engage in another, we tested whether foraging behavior and breeding behavior are correlated. Analyses were carried out over 1000 simulations testing for any effect (x is dependent on y or y is dependent on x) in Mesquite 3.02 (Maddison & Maddison, 2015) using the *correl* package. The evolution of foraging behavior and breeding behavior is correlated (*p* = .006, Pagel's correlation test; Pagel, 1994). This is unsurprising given that most social breeding species also forage socially (Table 1). Despite this correlation, we chose to analyze these traits separately because many solitary breeding species also forage socially. Additionally, there is much more variation in the manifestation of social breeding, with colony sizes varying from a single pair to 6,000 pairs (Brown et al., 2013; Turner, 2004), so the selective pressures of foraging socially and breeding socially may be quite different.

**Table 1:** Descriptive statistics for breeding and foraging behavior and results of phylogenetic *t*‐test based on 10,000 simulations for each trait in each social strategy. All measurements are in millimeters, and all *p*‐values have been adjusted for multiple tests using the method of Holm (1979)

	Breeding behavior					Foraging behavior				
	Solitary		Social		*p*‐Value	Pairs		Groups		*p*‐Value
	Mean	*SD*	Mean	*SD*		Mean	*SD*	Mean	*SD*	
Wing length	110.73	13.15	104.95	12.61	1	116.19	15.19	103.76	10.40	.040
Outer tail length	61.25	21.70	55.00	12.74	1	68.82	24.44	53.29	11.67	.047
Depth of tail fork	16.94	19.85	12.08	11.33	1	45.59	8.69	42.67	4.84	.053
Tarsus length	8.16	1.77	8.11	1.65	1	8.98	1.88	7.77	1.48	.053
Bill length	6.93	1.61	6.27	1.61	1	7.52	1.53	6.12	1.52	.050
Bill width	5.23	1.09	4.80	1.06	1	5.68	0.90	4.65	1.02	.040

### 2.3 Statistical analyses

We completed descriptive summary statistics for all morphological traits separated by behavioral category. The mean and standard deviation (*SD*) for each trait were calculated for species that breed solitarily or socially and forage in pairs or groups. *SD* was calculated rather than standard error (*SE*) to illustrate the difference in the variability of each morphological trait in social and nonsocial species. We performed *t*‐tests (using phylANOVA from phytools; Revell, 2011) between solitary and social categories and between pair and group foraging categories for each morphological trait to determine whether the mean trait values were significantly different between solitary and social species. We used the multiple testing correction of Holm (1979) to account for the many separate tests.

### 2.4 Phylogenetic analyses and model testing

Our phylogenetic analyses utilized the molecular phylogeny presented in Sheldon et al. (2005). The Sheldon et al. phylogeny contains sequence data for 75 of the 84 currently recognized species in the Hirundinidae (Clements et al., 2014; Dickinson, 2003). Of these 75 species, 72 are used in our analyses of breeding behavior and 73 are used in our analyses of foraging behavior. *Pseudochelidon sirintarae*,* Haplochelidon andecola*, and *Progne murphyi* were excluded from all analyses due to lack of morphological data. *Notiochelidon flavipes* was excluded from analyses of breeding behavior due of lack of data, but was included in foraging behavior analyses. To prevent inflation of the data at the tree tips, we excluded the following subspecies from all analyses: *Psalidoprocne pristoptera petiti*,* P. p. orientalis*, and *Hirundo rustica erythrogaster*. Instead, these species were represented in the analyses by the subspecies *P. p. holomelas* and *H. r. rustica*. These subspecies were chosen over the others because they were represented by more complete genetic sampling. The Sheldon et al. (2005) phylogeny included four outgroup species, which we excluded because they were not swallow species.

To illustrate how morphology clusters with social behavior, we generated phylomorphospaces for the group size of breeding colonies and the raw morphological measurements (Sidlauskas, 2008). Phylomorphospace plots were generated only for breeding behavior because breeding group size was more accurately available in the literature than foraging group size; foraging group size is often referenced vaguely in primary literature (e.g., large group, small group). Phylomorphospaces are raw data not subject to any direct analysis, and as such, they should be treated as exploratory analyses depicting the first‐order relationship of sociality and morphology.

Convergence is a difficult aspect of evolution to measure (Stayton, 2015). We used two different methods to first test for, and then quantify the strength of, convergence. First, we used four indices (C1–C4) that quantify how social lineages move through phenotypic space (Stayton, 2015). These indices use ancestral state reconstruction to look at the extent to which species have evolved greater similarity to one another. By comparing the distance between two tips relative to their distance at the point in the past where the two lineages were maximally dissimilar (C1), it is possible to test whether particular lineages are moving toward one another in phenotypic space. Likewise, the raw value of the difference between the maximum and extant distance between the two lineages (C2) can be scaled by either the total evolution (sum of squared ancestor‐to‐descendant changes) between the two lineages (C3) or the total evolution in the whole clade (C4). These metrics rely on ancestral state reconstruction of the various characters; however, these indices are the only reliable way to detect incomplete convergence in multidimensional space. We reconstructed ancestral states using the Bayesian implementation of the threshold model described by Revell (2014) with 2,000,000 generations, sampling every 2,000 generations, and discarding the first 10% as burn‐in. The threshold model is more appropriate as the liability can be interpreted as an unobserved continuous trait (such as blood hormone levels) and allows for different clades to have variable levels of lability. For instance, *Hirundo* includes both social and solitary species, while *Petrochelidon* is exclusively social, which would bias rate matrix approaches to ancestral state reconstruction. Significance was tested by simulating trait evolution 1,000 times along the phylogeny and determining what fraction of random‐trait evolution simulations show higher levels than the observed data.

Another metric of convergence, which does not rely on ancestral state reconstruction, is the Wheatsheaf index (Arbuckle et al., 2014). The Wheatsheaf index compares the mean distance in phenotypic space between social species to the overall average distance between all pairs of species and scales those comparisons by the phylogenetic variance–covariance matrix. Unlike Stayton's (2015) indices, the Wheatsheaf index cannot test for incomplete convergence, nor does it test for the presence of convergence per se. Rather, it quantifies the strength of convergence among taxa and, by permuting the tip data, tests whether or not that strength is significant relative to the overall evolution of the clade. One major advantage of the Wheatsheaf index is that it makes no assumptions about the ancestral states; it is simply a phylogenetically corrected statistic of distances between taxa.

Finally, we also used the package l1ou, a model‐based approach to detecting convergence which employs LASSO (least absolute shrinkage and selector operator) to determine the optimal number of selective regimes in a phylogeny (Khabbazian, Kriebel, Rohe, & Ané, 2016). l1ou paints a phylogeny with different Ornstein–Uhlenbeck models (OU; Hansen, 1997; Butler & King, 2004; Beaulieu et al. 2012) to determine how many different selection regimes are needed to explain the data and then tries to collapse those regimes together. Convergence is indicated by either identical (collapsed) or very similar sets of OU parameters in distantly related taxa. This method requires no prespecification of taxa nor the number or location of rate shifts. All inferred heterogeneity and the positions of transitions are automatically detected. However, this approach is fully model‐based, and subject to all the perils of OU models in general (e.g., see Cooper, Thomas, Venditti, Meade, & Freckleton, 2016), and only allows for shifts in the theta value. In our study, it is primarily useful in demonstrating nonhomogenous evolutionary dynamics.

All calculations, graphs, and simulations were completed in R 3.1.0 (R Core Team 2014), using functions from the packages “vegan,” “ape,” “phytools,” “l1ou,” “MASS,” “msm,” and their dependencies (Beaulieu & O'Meara, 2014; Jackson, 2011; Khabbazian et al., 2016; Oksanen et al., 2013; Paradis, Claude, & Strimmer, 2004; Revell, 2011). All code, data, and model fitting outputs are archived at Dryad (doi:10.5061/dryad.m07t1).

## 3 Results

### 3.1 Descriptive statistics

Most morphological traits, whether in solitary or social categories of breeding and foraging behavior, have similar mean values (Table 1). For breeding behavior, only the mean bill length and width are significantly smaller in social than solitary species (two‐tailed *t*‐test, Table 1). For foraging behavior, four morphological traits (outer tail length, depth of tail fork, tarsus length, and bill width) are significantly smaller in group foragers compared to solitary species (two‐tailed *t*‐test, Table 1). While not all morphological traits differ between solitary and social species, the mean values of the traits of social breeders and foragers generally have smaller standard deviations than that of nonsocial species (three of six traits for breeding behavior and five of six for foraging behavior; Table 1).

The low external morphological variation in social species is illustrated by the phylomorphospace plots of maximum breeding group size (Figure 2). Species that exhibit solitary behavior fill a broader morphological space than species that exhibit social behavior; the small morphological space filled by socially breeding species remains the same despite variation in group size.

### 3.2 Repeated evolution of social behaviors

Of the 72 species included in our analyses of breeding behavior, 33 species were categorized as solitary and 49 were categorized as social. Of the 73 species included in our foraging dataset, there are 20 species that forage either solitarily or in pairs while 53 forage in groups. Transitions in behavior were common, but unevenly distributed across the phylogeny. Some genera, such as *Hirundo* and *Progne,* have multiple transitions to and from social behavior, while older genera like *Petrochelidon* show no heterogeneity at all. The ancestral swallow is well‐supported as a social breeder and forager in our analyses based on the threshold model.

### 3.3 Testing for and quantifying convergence

**Figure 3:**
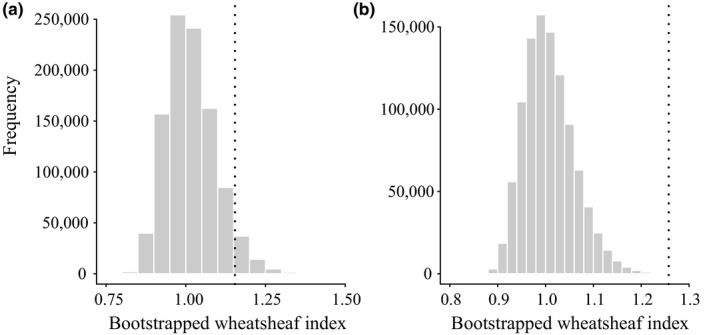
Strength of convergence as measured by the Wheatsheaf index for social breeding (a) and foraging (b) species. Histograms represent the distribution of the Wheatsheaf index for 1,000,000 randomizations of the data, and the dashed lines show the value of the Wheatsheaf index for the observed data

**Figure 4:**
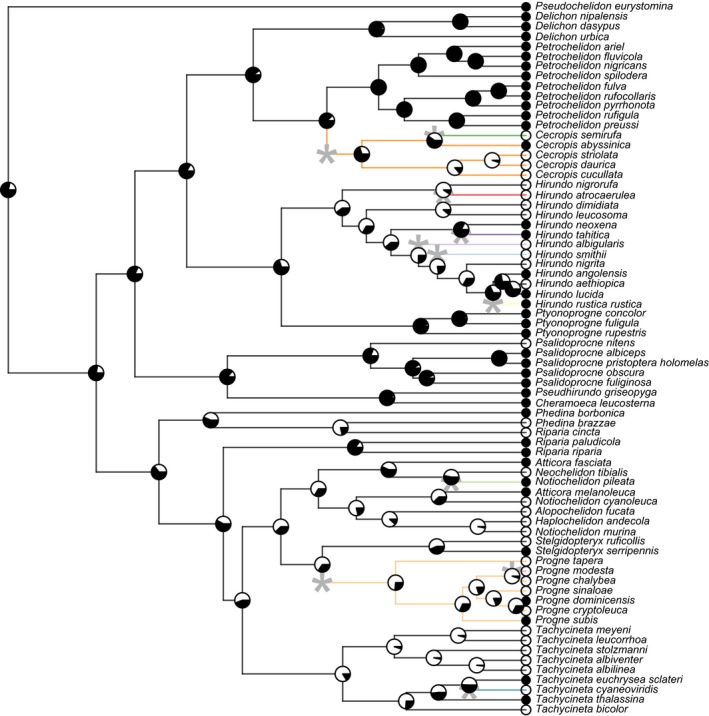
Ancestral reconstruction of breeding behavior using the threshold model and evolutionary regimes using l1ou. White icons denote solitary species, while black indicates social species, and pie charts at each node show the posterior probability of each character state at that node. Edges are colored by regime, and asterisks denote the location of regime shifts

**Figure 5:**
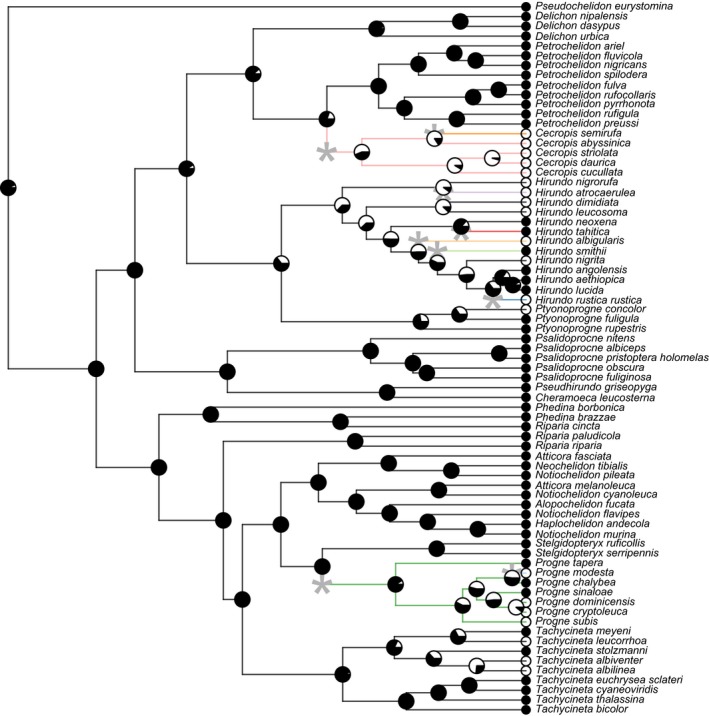
Ancestral reconstruction of foraging behavior using the threshold model and evolutionary regimes using l1ou. As in Figure 4, white icons denote solitary species, while black indicates social species, and pie charts at each node show the posterior probability of each character state at that node. Edges are colored by regime, and asterisks denote the location of regime shifts

Both socially foraging and breeding swallow species converged significantly according to the indices of Stayton (2015). Social swallows show 22% convergence in the morphological traits measured, which represents about 10% of the overall phenotypic evolution of the social species and 1% of morphological evolution in all swallows. This amount of convergence was significant in both foraging (*p* = .007) and breeding (*p* = .002) based on 1,000 Brownian motion simulations. Likewise, the Wheatsheaf index shows strong convergence in both social breeders and foragers, although only the strength of convergence in social foragers is significant (*p* < .01; Figure 3). l1ou analysis (Khabbazian et al., 2016) found evidence for 13 shifts in breeding behavior (Figure 4) and 11 shifts in foraging behavior (Figure 5). Many of these shifts in evolutionary regimes occurred on branches where transitions in social behavior occurred. l1ou only allows for changes in the trait optimum, making it difficult to compare directly with other methods. However, the results clearly indicate heterogeneity in swallow phenotypic evolution.

## 4 Discussion

Sociality in the Hirundinidae appears to be associated with changes in morphology, with social species exhibiting smaller, more constrained morphological traits than their nonsocial relatives (Figure 2). This pattern can be explained in four ways. It may be the result of (1) small sample size, (2) phylogenetic autocorrelation in which one ancestral swallow became social and its descendants inherited a similar morphology, (3) constraint on social species from something other than behavior, such as habitat, or (4) direct selection on morphology driven by sociality, by increasing competition for shared resources and promoting convergence. Social breeding and social foraging have been acquired and lost repeatedly in the Hirundinidae with significant consequences for the evolution of external morphology. The repeated shifts between social and nonsocial behavior in the Hirundinidae reduce support for the first two explanations, as convergence upon morphology had to have occurred multiple times, and could not have come from a single common ancestor. The patterns of lower variation and higher convergence in social, relative to solitary, swallows were observed in the raw data (Figure 2) and supported by a variety of analyses. Comparison of the evolutionary trajectories of social and solitary lineages strongly support convergence in social species, as does a simple, phylogenetically corrected calculation of how clumped social species are in morphospace.

All swallows are aerial insectivores suggesting all species must be near a similar morphological optimum to allow for aerial foraging (Turner, 2004). Convergence occurs in both socially breeding and foraging species, although the convergence is stronger in socially foraging species. Solitarily foraging species typically consume larger, more solitary insect prey than do social foraging species, which often feed on mass insect emergences (Bryant & Turner, 1982; Turner, 1982; Quinney & Ankney, 1985; Brown & Brown, 1996; Kopij, 2000; Chișamera & Manole, 2005; Fernandes, Cruz, & Rodrigues, 2007; Boukhemza‐Zemmouri, Farhi, Sahnoun, & Moukhemza, 2013; Orlowski & Karg, 2013; M. B. Brown, pers. obs.). While this difference in prey types may suggest ecology to be an important driver of morphological changes, swallows may only be able to specialize on small ephemeral insects when sharing information within a flock, suggesting a combination of social and ecological behaviors alter the optimal morphologies in different swallows. Avoiding collisions as multiple individuals feed on the same emergent insect swarm may necessitate a particular acrobatic morphology and so may explain our results.

Both social foraging and social breeding require agile flight. Species that require aerodynamic maneuverability tend to have proportionately shorter tails and wings, which provide high lift to drag ratios, whereas species that require less agile flight typically have longer tails (Brown & Brown, 2013; Evans and Thomas, 1992; Thomas & Balmford, 1995). Wing length and outer tail length are significantly shorter in group foraging species than in pair foraging species. Depth of tail fork is also smaller in group foraging species than in pair foraging species; however, this result is marginally significant. The shorter outer tail length and shallower depth of tail fork result in a more square‐shaped tail in group foraging species. These patterns hold for breeding behavior but are not statistically significant. Separate from social foraging, agile flight in social breeders may be advantageous by reducing the likelihood of collisions at colony sites where many birds are moving in and out of nests.

We also see significantly reduced bill length and width in socially foraging species, resulting in relatively smaller bills, a pattern which again holds for breeding behavior but which is not statistically significant. The reasons for constraint in these traits may be twofold. First, and most importantly, bill size influences foraging success. All members of the Hirundinidae consume insects they capture in flight. As noted above, insects consumed by nonsocial species (e.g., barn swallow, *Hirundo rustica*) are typically larger in size compared to those consumed by social species (Bryant & Turner, 1982; Turner, 1982; Quinney & Ankney, 1985; Brown & Brown, 1996; Chișamera & Manole, 2005; Fernandes et al., 2007; Boukhemza‐Zemmouri et al., 2013; Orlowski & Karg, 2013; M. B. Brown, pers. obs.). Additionally, most of the insects consumed by social species are found in aggregations (e.g., mating swarms, mass emergences, local convection currents) and birds foraging in groups may be more able to locate and exploit them as a food resource. This has been shown in cliff swallows (*Petrochelidon pyrrhonota*, Figure 1), where colonies act as information centers and large colonies facilitate tracking of ephemeral insects (Brown, 1988). As social species specialize in foraging on small ephemeral insects, large bills may be selected against. Second, bill size may influence the construction of nest structures in social species (Winkler & Sheldon, 1993). Species that form the largest colonies (e.g., *Petrochelidon* sp.) all build mud retorts that require birds to collect, carry, and adhere mud to form their nests using their beaks, and perhaps, smaller bills influences transport and application of mud. However, similar mud‐type nests are found in a few of the solitary species (e.g., *Cecropis* sp., open mud cups, *Hirundo* sp.; Winkler & Sheldon, 1993; Turner, 2004), so we feel more weight should be given to the foraging specialization hypothesis.

Our analyses suggest there is a consistent morphological “solution” to being social in the Hirundinidae; that is, social swallows have converged on only one morphological type. This is supported by within species studies on cliff swallows which show no morphological difference between swallows that occupy large colonies or small colonies, even though colony choice is heritable for first year colony preference (Brown & Brown, 1996, 2000; Roche et al., 2011). Aside from Winkler and Sheldon's (1993) study demonstrating a link between nest morphology and degree of sociality in swallows, this is the first study illustrating a link between sociality and morphology in birds of which we are aware.

We have shown that morphological evolution is associated with changes in social structure, both in breeding and in foraging. As we are quantifying social foraging in addition to breeding, it is obvious that the behaviors we observe are linked to ecological behaviors, such as the type and size of insects preyed upon. However, in swallows it is a change in social behavior that is changing ecological behaviors and both aspects of behavior influence morphology. As such, social behavior is the ultimate cause of these changes; however, our data also suggest that social behavior is the proximate driver as well. While the evolution of certain nesting structures (e.g., mud retorts) either facilitated or followed the evolution of extremely large colonies (Winkler & Sheldon, 1993), each nest type is found in both solitary and social breeding species. Although foraging habitat (open or closed) influences wing and tail morphology in other aerial insect feeders (e.g., bats; Kalcounis & Brigham, 1995), swallows and martins are generally all found to forage in open habitat. This consistent foraging preference for open habitats in swallows suggests we would not expect to see a shift toward greater maneuverability unless driven by some other selective pressure. Finally, it is possible that sexual selection may influence the evolution of morphologies observed in swallows. However, only one species, *Hirundo smithii*, has extremely dimorphic morphological traits, with males exhibiting long outer tail “streamers.” While sexual selection certainly results in dimorphic morphology in some swallow species (Møller 1992; Møller and Birkhead 1992), males and females generally exhibit similar morphologies and, except for in the case of *H. smithii*, we feel averaging morphological measurements across sexes was sufficient to compensate for this variation.

We have shown that sociality produces morphological convergence in the Hirundinidae. We see many transitions between solitary and social breeding behavior as well as between pair and group foraging behavior, but the same morphology evolves every time a species becomes social. This suggests that social behavior in the Hirundinidae is successful only within a single morphological niche space. Further studies in taxa with both social and nonsocial behaviors may inform whether the evolution of sociality consistently constrains morphological evolution or if, in some cases, it promotes morphological diversity. More studies are necessary to understand the potential for social behavior to alter the morphological evolutionary trajectory of species.

## Conflict of Interest

None declared.

## Data Accessibility

The datasets and code used and described in this article are archived at Dryad (doi:10.5061/dryad.m07t1).

## Data Archiving

Social and morphological data and R code utilized for data analysis have been submitted as supplementary material associated with this manuscript.
