# Supplementary material for: Convergent evolution in social swallows (Aves: Hirundinidae)
Source: Ecol Evol. 2016 Dec 20;7(2):550–60. doi: 10.1002/ece3.2641 (PMC5243784; doi:10.1002/ece3.2641)
Supplement: Supplementary file 1 [file ECE3-7-550-s001.docx]

**Supplementary Material**

**Allison E. Johnson, Jonathan Mitchell and Mary Bomberger Brown. Convergent evolution in social swallows (Aves: Hirundinidae)**

**Appendix S1. Morphological and behavioral data for Hiruninidae species**

**Appendix S1. Morphological and behavioral data for Hirundinidae species**

**Table A1.** Number of specimens measured for each taxa and morphological measurements (including wing length, tail length, depth of tail fork, bill length and bill width) averaged over all specimens measured for each taxa.

| Species | Subspecies | # males | # females | Total # specimens (unknown sex included) | Wing (mm) | Tail (mm) | Depth of tail fork (mm) | Tarsus (mm) | Bill length (mm) | Bill width (mm) |
| --- | --- | --- | --- | --- | --- | --- | --- | --- | --- | --- |
| *Aplochelidon fucata* |  | 3 | 2 | 5 | 99.1 | 43.5 | 4.3 | 5.91 | 4.82 | 3.42 |
| *Atticora fasciata* |  | 5 | 5 | 10 | 95.2 | 66.1 | 20.9 | 5.85 | 5.21 | 3.88 |
| *Atticora melanoleuca* |  | 2 | 1 | 7 | 89.9 | 65.1 | 16.8 | 6.39 | 4.74 | 3.56 |
| *Cecropis abyssinica* |  | 5 | 5 | 10 | 106.2 | 77.8 | 31.0 | 10.11 | 6.14 | 5.10 |
| *Cecropis cucullata* |  | 5 | 4 | 9 | 122.3 | 91.4 | 39.4 | 10.42 | 6.91 | 5.66 |
| *Cecropis daurica* |  | 5 | 5 | 10 | 114.9 | 79.4 | 20.9 | 9.18 | 6.50 | 5.75 |
| *Cecropis semirufa* |  | 5 | 5 | 10 | 119.0 | 106.8 | 62.3 | 10.85 | 6.88 | 5.89 |
| *Cecropis striolata* |  | 4 | 5 | 10 | 120.9 | 80.1 | 21.0 | 9.32 | 7.45 | 5.70 |
| *Cheramoeca leucosterna* |  | 0 | 2 | 5 | 101.3 | 71.0 | 29.8 | 9.67 | 5.40 | 4.63 |
| *Delichon dasypus* |  | 6 | 4 | 10 | 102.8 | 43.8 | 1.8 | 7.65 | 5.32 | 4.42 |
| *Delichon nipalensis* |  | 3 | 3 | 6 | 88.2 | 38.3 | 1.6 | 7.20 | 5.07 | 3.95 |
| *Delichon urbica* |  | 5 | 5 | 10 | 108.7 | 56.9 | 11.1 | 8.56 | 5.79 | 5.14 |
| *Haplochelidon andecola* |  | 2 | 3 | 5 | 109.3 | 52.1 | 3.9 | 8.82 | 6.05 | 4.77 |
| *Hirundo aethiopica* |  | 2 | 6 | 8 | 104.3 | 55.7 | 16.7 | 7.73 | 7.06 | 5.34 |
| *Hirundo albigularis* |  | 3 | 2 | 5 | 130.3 | 71.6 | 31.8 | 8.07 | 8.27 | 5.12 |
| *Hirundo angolensis* |  | 5 | 4 | 9 | 114.5 | 55.8 | 14.1 | 7.60 | 7.69 | 5.73 |
| *Hirundo atrocaerulea* |  | 5 | 0 | 5 | 107.4 | 122.8 | 75.8 | 7.87 | 6.75 | 5.11 |
| *Hirundo dimidiata* |  | 2 | 3 | 5 | 98.3 | 52.6 | 12.6 | 6.86 | 6.22 | 5.15 |
| *Hirundo leucosoma* |  | 2 | 1 | 4 | 95.5 | 41.9 | 9.1 | 6.79 | 5.95 | 4.40 |
| *Hirundo lucida* |  | 2 | 2 | 4 | 110.6 | 57.6 | 19.3 | 7.48 | 7.23 | 5.41 |
| *Hirundo nigrita* |  | 4 | 5 | 9 | 107.2 | 38.6 | 4.4 | 8.21 | 7.47 | 5.50 |
| *Hirundo rustica* | *rustica* | 5 | 5 | 10 | 119.1 | 93.4 | 51.0 | 8.07 | 7.05 | 5.54 |
| *Hirundo smithii* |  | 5 | 5 | 10 | 112.3 | 103.5 | 66.9 | 7.99 | 6.91 | 4.97 |
| *Hirundo tahitica* |  | 5 | 3 | 8 | 107.2 | 44.9 | 8.3 | 7.75 | 8.10 | 6.35 |
| *Hirundo nigrorufa* |  | 1 | 1 | 2 | 102.0 | 52.5 | 15.5 | 7.42 | 6.07 | 5.10 |
| *Hirundo neoxena* |  | 1 | 2 | 7 | 108.7 | 68.1 | 22.1 | 8.59 | 6.56 | 4.69 |
| *Neochelidon tibialis* | *grisiventris* | 5 | 4 | 9 | 91.2 | 46.4 | 6.1 | 5.08 | 4.89 | 3.87 |
| *Notiochelidon cyanoleuca* |  | 5 | 5 | 10 | 93.3 | 44.9 | 9.1 | 5.19 | 4.60 | 3.50 |
| *Notiochelidon flavipes* |  | 10 | 5 | 15 | 91.0 | 47.8 | 8.5 | 7.33 | 4.30 | 2.69 |
| *Notiochelidon murina* |  | 4 | 5 | 9 | 110.4 | 60.9 | 9.6 | 6.61 | 5.20 | 3.64 |
| *Notiochelidon pileata* |  | 1 | 0 | 1 | 98.0 | 55.0 | 24.0 | 7.65 | 5.24 | 4.02 |
| *Petrochelidon fluvicola* |  | 4 | 5 | 9 | 91.1 | 39.8 | 3.1 | 7.56 | 5.68 | 4.62 |
| *Petrochelidon fulva* |  | 4 | 5 | 9 | 99.3 | 41.7 | 2.4 | 10.43 | 6.33 | 5.21 |
| *Petrochelidon nigricans* |  | 4 | 5 | 9 | 105.0 | 47.9 | 5.2 | 8.40 | 6.13 | 4.89 |
| *Petrochelidon preussi* |  | 4 | 3 | 7 | 94.0 | 47.3 | 7.0 | 8.51 | 5.53 | 4.27 |
| *Petrochelidon pyrrhonota* |  | 5 | 4 | 9 | 109.2 | 48.7 | 2.5 | 10.43 | 6.67 | 5.37 |
| *Petrochelidon rufigula* |  | 2 | 0 | 2 | 96.5 | 48.8 | 5.3 | 8.56 | 6.72 | 5.34 |
| *Petrochelidon spilodera* |  | 1 | 0 | 1 | 104.0 | 47.5 | 3.5 | 8.42 | 7.25 | 6.04 |
| *Petrochelidon rufocollaris* |  | 1 | 1 | 2 | 98 | 44 | 2 | 8.035 | 6.28 | 4.97 |
| *Petrochelidon ariel* |  | 2 | 1 | 3 | 87.25 | 41.5 | 3.5 | 7.68625 | 5.32 | 4.36 |
| *Phedina borbonica* |  | 1 | 0 | 1 | 111.0 | 48.5 | 3.5 | 10.78 | 7.74 | 5.39 |
| *Phedina brazzae* |  | 0 | 1 | 1 | 97.0 | 45.0 | 2.0 | 7.85 | 5.61 | 4.00 |
| *Progne chalybea* |  | 5 | 5 | 10 | 124.6 | 58.8 | 13.4 | 10.40 | 10.22 | 7.16 |
| *Progne cryptoleuca* |  | 0 | 2 | 3 | 142.7 | 71.3 | 14.0 | 12.30 | 10.83 | 7.70 |
| *Progne dominicensis* |  | 4 | 2 | 6 | 141.4 | 67.8 | 10.1 | 12.38 | 10.50 | 7.29 |
| *Progne sinaloae* |  | 3 | 0 | 3 | 132.5 | 68.5 | 10.5 | 12.40 | 10.20 | 7.34 |
| *Progne modesta* |  | 1 | 0 | 1 | 122.0 | 60.0 | 16.0 | 10.14 | 8.22 | 6.77 |
| *Pronge subis* |  | 5 | 5 | 10 | 142.3 | 66.2 | 14.4 | 11.61 | 10.44 | 7.38 |
| *Progne tapera* |  | 5 | 5 | 10 | 132.6 | 62.7 | 6.7 | 10.37 | 10.52 | 6.64 |
| *Psalidoprocne albiceps* |  | 5 | 5 | 10 | 99.3 | 63.9 | 15.2 | 6.65 | 5.23 | 4.11 |
| *Psalidoprocne fuligosa* |  | 0 | 1 | 1 | 88.5 | 52.0 | 13.0 | 6.93 | 4.12 | 3.72 |
| *Psalidoprocne nitens* |  | 5 | 4 | 9 | 92.3 | 45.6 | 1.3 | 6.64 | 4.82 | 3.72 |
| *Psalidoprocne obscura* |  | 2 | 3 | 5 | 94.2 | 71.2 | 28.0 | 6.49 | 4.64 | 3.64 |
| *Psalidoprocne pristoptera* | *holomelas* | 4 | 5 | 9 | 104.3 | 74.6 | 26.3 | 6.67 | 5.21 | 4.27 |
| *Pseudhirundo griseopyga* |  | 5 | 5 | 10 | 92.7 | 71.2 | 27.8 | 5.34 | 5.09 | 3.50 |
| *Pseudochelion eurystomia* |  | 1 | 1 | 2 | 117.5 | 42.0 | 2.0 | 11.02 | 10.64 | 7.12 |
| *Ptyonoprogne concolor* |  | 5 | 4 | 9 | 108.2 | 44.8 | 3.0 | 7.84 | 6.57 | 4.59 |
| *Ptyonoprogne fuligula* |  | 5 | 4 | 9 | 110.9 | 44.9 | 1.9 | 8.13 | 6.87 | 5.16 |
| *Ptyonoprogne rupsetris* |  | 5 | 5 | 10 | 127.4 | 50.2 | 2.1 | 8.60 | 7.30 | 5.48 |
| *Riparia cincta* |  | 5 | 5 | 10 | 124.2 | 55.9 | 2.9 | 10.38 | 7.98 | 6.58 |
| *Riparia paludicola* |  | 5 | 4 | 9 | 100.6 | 46.2 | 2.5 | 7.94 | 5.64 | 4.06 |
| *Riparia riparia* |  | 5 | 5 | 10 | 99.9 | 46.8 | 4.1 | 7.61 | 5.82 | 4.45 |
| *Stelgidopteryx ruficollis* |  | 5 | 5 | 10 | 104.6 | 48.6 | 2.9 | 7.57 | 7.13 | 5.04 |
| *Stelgidopteryx serripennis* |  | 5 | 5 | 10 | 101.6 | 45.3 | 2.3 | 7.68 | 6.38 | 4.45 |
| *Tachycineta albilinea* |  | 5 | 5 | 10 | 95.8 | 40.1 | 4.4 | 6.58 | 6.79 | 5.28 |
| *Tachycineta albiventer* |  | 5 | 5 | 10 | 104.3 | 44.6 | 7.6 | 7.91 | 7.99 | 5.53 |
| *Tachycineta bicolor* |  | 5 | 5 | 10 | 111.6 | 48.8 | 7.4 | 7.71 | 5.66 | 4.49 |
| *Tachycineta cyaneoviridis* |  | 2 | 4 | 6 | 108.1 | 58.9 | 12.7 | 6.99 | 6.19 | 5.21 |
| *Tachycineta euchrysea* | *sclateri* | 5 | 4 | 9 | 110.2 | 46.1 | 7.7 | 6.29 | 4.57 | 3.64 |
| *Tachycineta leucopyga* |  | 5 | 2 | 7 | 109.5 | 48.4 | 4.5 | 8.36 | 6.46 | 4.97 |
| *Tachycineta leucorrhoa* |  | 2 | 4 | 6 | 115.6 | 47.6 | 5.4 | 7.29 | 6.66 | 5.61 |
| *Tachycineta thalassina* |  | 5 | 5 | 10 | 110.8 | 42.3 | 5.5 | 6.75 | 4.79 | 3.58 |
| *Tachycineta stolzmanni* |  | 1 | 0 | 1 | 95.0 | 48.5 | 3.5 | 6.97 | 5.24 | 4.46 |

**Table A2.** Breeding behavior and foraging behavior for all taxa. Breeding behavior was categorized as solitary (only a single breeding pair) or social (groups of 2 or more breeding pairs) and foraging behavior was categorized as pair (only the breeding pair forages together) or group (foraging in groups of two or more breeding pairs). Sources for each species can be found in the text following Table A2.

| Species | Subspecies | Breeding behavior | Foraging behavior | Sources |
| --- | --- | --- | --- | --- |
| *Aplochelidon fucata* |  | Solitary | Group | 1 |
| *Atticora fasciata* |  | Social | Group | 2 |
| *Atticora melanoleuca* |  | Social | Group | 2 |
| *Cecropis abyssinica* |  | Social | Pairs | 3 |
| *Cecropis cucullata* |  | Solitary | Pairs | 3 |
| *Cecropis daurica* |  | Solitary | Pairs | 3 |
| *Cecropis semirufa* |  | Solitary | Pairs | 3 |
| *Cecropis striolata* |  | Solitary | Pairs | 3 |
| *Cheramoeca leucosterna* |  | Social | Group | 3 |
| *Delichon dasypus* |  | Social | Group | 2 |
| *Delichon nipalensis* |  | Social | Group | 3 |
| *Delichon urbica* |  | Social | Group | 3 |
| *Haplochelidon andecola* |  | Solitary | Group | 3 |
| *Hirundo aethiopica* |  | Solitary | Group | 3 |
| *Hirundo albigularis* |  | Solitary | Pairs | 3, 4 |
| *Hirundo angolensis* |  | Social | Group | 3 |
| *Hirundo atrocaerulea* |  | Solitary | Pairs | 5 |
| *Hirundo dimidiata* |  | Solitary | Pairs | 3, 6 |
| *Hirundo leucosoma* |  | Solitary | Pairs | 3 |
| *Hirundo lucida* |  | Social | Group | 2 |
| *Hirundo nigrita* |  | Solitary | Pairs | 3 |
| *Hirundo rustica* | *rustica* | Social | Pairs | 3, 7, 8 |
| *Hirundo smithii* |  | Solitary | Group | 3 |
| *Hirundo tahitica* |  | Social | Group | 9 |
| *Hirundo nigrorufa* |  | Solitary | Pairs | 3 |
| *Hirundo neoxena* |  | Social | Group | 10 |
| *Neochelidon tibialis* | *grisiventris* | Solitary | Group | 11 |
| *Notiochelidon cyanoleuca* |  | Solitary | Group | 12 |
| *Notiochelidon flavipes* |  |  | Group | 13 |
| *Notiochelidon murina* |  | Solitary | Group | 14 |
| *Notiochelidon pileata* |  | Social | Group | 15, 16 |
| *Petrochelidon fluvicola* |  | Social | Group | 3 |
| *Petrochelidon fulva* |  | Social | Group | 17 |
| *Petrochelidon nigricans* |  | Social | Group | 3 |
| *Petrochelidon preussi* |  | Social | Group | 3 |
| *Petrochelidon pyrrhonota* |  | Social | Group | 3, 18 |
| *Petrochelidon rufigula* |  | Social | Group | 3 |
| *Petrochelidon spilodera* |  | Social | Group | 3 |
| *Petrochelidon rufocollaris* |  | Social | Group | 19 |
| *Petrochelidon ariel* |  | Social | Group | 3, 20 |
| *Phedina borbonica* |  | Social | Group | 3 |
| *Phedina brazzae* |  | Solitary | Group | 3 |
| *Progne chalybea* |  | Solitary | Group | 3 |
| *Progne cryptoleuca* |  | Solitary | Pairs | 3, 21 |
| *Progne dominicensis* |  | Social | Pairs | 2, 3 |
| *Progne sinaloae* |  | Solitary | Group | 3 |
| *Progne modesta* |  | Solitary | Pairs | 22 |
| *Pronge subis* |  | Social | Pairs | 3, 23 |
| *Progne tapera* |  | Solitary | Group | 21 |
| *Psalidoprocne albiceps* |  | Social | Group | 2, 3 |
| *Psalidoprocne fuligosa* |  | Social | Group | 3 |
| *Psalidoprocne nitens* |  | Solitary | Group | 2, 3 |
| *Psalidoprocne obscura* |  | Social | Group | 3 |
| *Psalidoprocne pristoptera* | *holomelas* | Social | Group | 3 |
| *Pseudhirundo griseopyga* |  | Social | Group | 3 |
| *Pseudochelion eurystomia* |  | Social | Group | 2, 3, 24 |
| *Ptyonoprogne concolor* |  | Social | Pairs | 2, 3 |
| *Ptyonoprogne fuligula* |  | Social | Group | 3 |
| *Ptyonoprogne rupsetris* |  | Social | Group | 3 |
| *Riparia cincta* |  | Solitary | Group | 3 |
| *Riparia paludicola* |  | Social | Group | 3 |
| *Riparia riparia* |  | Social | Group | 25, 26 |
| *Stelgidopteryx ruficollis* |  | Solitary | Group | 3 |
| *Stelgidopteryx serripennis* |  | Social | Group | 3 |
| *Tachycineta albilinea* |  | Solitary | Pairs | 27 |
| *Tachycineta albiventer* |  | Solitary | Pairs | 28, 29 |
| *Tachycineta bicolor* |  | Solitary | Group | 30, 31 |
| *Tachycineta cyaneoviridis* |  | Solitary | Group | 3 |
| *Tachycineta euchrysea* | *sclateri* | Social | Group | 32, 33, 34 |
| *Tachycineta leucopyga* |  | Solitary | Group | 35, 36 |
| *Tachycineta leucorrhoa* |  | Solitary | Pairs | 37, 3 |
| *Tachycineta thalassina* |  | Social | Group | 38 |
| *Tachycineta stolzmanni* |  | Solitary | Group | 2, 39, 40 |

References

1. Belton, W. 1985. Birds of Rio Grande do Sul, Brazil Part 2. Formicariidae through Corvidae. Bull. Amer. Mus. Nat. Hist. 180: 1-241.
2. Turner, A., and C. Rose. 1989. Swallows and martins: an identification guide and handbook. Boston, MA: Houghton Mifflin.
3. Turner, A. K. 2004. Family Hirundinidae (swallows and martins). In del Hoyo J, Elliot A, Christie DA, editors. Handbook of the Birds of the World Vol. 9. Barcelona, Spain: Lynx Edicions.
4. Jackson, H. D., and C. Spottiswoode. 2004. Breeding biology and taxonomy of the red-breasted swallow, Hirundo semirufa, in Zimbabwe. Ostrich 75: 5-10.
5. Evans, S. W., and H. Bouwman. 2009. Habitat selection by blue swallows Hirundo atrocaerulea Sundavall, 1850 breeding in South Africa and its implications for conservation. Afr, J. Ecol. 48: 871-879.
6. Schmidt, R. K. 1959. Notes on the pearl-breasted swallow Hirundo dimidiate in the south-western Cape. Ostrich 30:155-158.
7. Snapp, B. D. 1976. Colonial breeding in the barn swallow (Hirundo rustica) and its adaptive significance. Condor 78: 471-480.
8. Møller, A. P. 1987. Advantages and disadvantages of coloniality in the swallow, Hirundo rustica. Animal Behaviour 35: 819-832.
9. Hails, C. J. 1984. The breeding biology of the Pacific swallow Hirundo tahitica in Malaysia. Ibis 126: 198-211.
10. Marchant, S., and P. J. Fullagar. 1982. Nest records of the welcome swallow. Emu 86: 66-74.
11. Wetmore, A., R. F. Pasquier, and S. L. Olson. 1984. Passeriformes: Dendrocolaptidae (woodcreepers) to Oxyruncidae (sharpbills), The Birds of the Republic of Panama, Vol 4. Smithsonian Miscellaneous Collections 150(4). Washington D. C: Smithsonian Institution.
12. Skutch, A. F. 1952. Life history of the blue-and-white swallow. Auk 69: 392-406.
13. Parker, T. A., and J. P. O’Neill. 1980. Notes on little known birds of the upper Urubamba Valley, Southern Peru. Auk 97: 167-176.
14. Greeney, H. F., P. R. Martin, R. A. Gelis, A. Solano-Ugalde, F. Bonier, B. Freeman, and E. T. Mille. 2011. Notes on the breeding of high-Andean birds in northern Ecuador. Bull. Brit. Ornithol. Club 131: 24-31.
15. Baepler, D. H. 1962. The avifauna of the Soloma region in Huehuetenango Guatemala. Condor 64: 140-153.
16. Marshall, J. T. 1943. Additional information concerning the birds of El Salvador. Condor 45: 21-33.
17. Selander, R. K., and J. K. Baker. 1957. The cave swallow in Texas. Condor 59: 345-363.
18. Brown, C. R., and M. B. Brown. 1996. Coloniality in the cliff swallow: the effect of group size on social behavior. Chicago, IL: University of Chicago Press.
19. Cisneros-Heredia, D. F. 2006. Notes on breeding, behaviour and distribution of some birds in Ecuador. Bull. Brit. Ornithol. Club 126: 153-164.
20. Santema, P., S. C. Griffith, N. E. Langmore, J. Komedeur, and M. J. L. Magrath. 2009. Does foraging efficiency vary with colony size in the fairy-martin Petrochelidon ariel? J. Avian Biol. 40: 57-66.
21. Morton, E. S., L. Forman, and M. Braun. 1990. Extrapair fertilizations and the evolution of colonial breeding in purple martins. Auk 107: 275-283.
22. Gifford, E. T. 1919. Field notes on the land birds of the Galapagos Islands and of Cocos Island. Proc. Cali. Acad. Sci. 2: 189-258.
23. Allen, R. W., and M. M. Nice. 1952. A study of the breeding biology of the purple martin (Progne subis). Am. Mid. Nat. 47: 606-665.
24. Chapin, J. P. 1953. The birds of the Belgian Congo part 3. Bulletin of the Am. Mus. Nat. Hist. 75: 9-821.
25. Hoogland, J. L., and P. W. Sherman. 1976. Advantages and disadvantages of bank swallow (Riparia riparia) coloniality. Ecol. Monog. 46: 33-58. (doi:10.2307.1942393)
26. Garrison, B. A. 1999. Bank swallow (Riparia riparia). In: Poole A, Gill F editors. The Birds of North America, No. 414.Philadelphia, PA: The Birds of North America, Inc.
27. Dyrcz, A. 1984. Breeding biology of the mangrove swallow (Hirundo atrocaerulea). Nyala 12: 27-30.
28. Greeney, H. F., and P. A. M. Merino. 2006. Notes on breeding birds from the Cuyabeno Faunistic Reserve in North Eastern Ecuador. Boletín SAO 16: 46-54.
29. Ricklefs, R. E. 1971. Foraging behavior of mangrove swallows at Barro Colorado Island. Auk 88: 635-651.
30. Muldal, A., H. L. Gibbs, R. J. Robertson. 1985. Preferred nest spacing of an obligate cavity-nesting bird, the tree swallow. Condor 87: 356-363.
31. Wheelwright, N. T., J. Leary, and C. Fitzgerald. 1991. The costs of reproduction in tree swallows (Tachycineta bicolor). Can. J Zool. 69: 2540-2547.
32. Townsend, J. M., E. Garrido, and D. A. Mejia. 2008. Nests and nesting behavior of golden swallow (Tachycineta euchrysea) in abandoned bauxite mines in the Dominican Republic. Wilson J. Ornithol. 120: 867-871.
33. Graves, G. R. 2013. Historical decline and probable extinction of the Jamaican golden swallow Tachycineta euchrysea euchrysea. Bird Conserv. Int. 24: 239-251. (doi:10.1017/S095927091300035X)
34. Dod, A. S. 1992 Endangered and endemic birds of the Dominican Republic. Fort Bragg, California: Cypress House Press.
35. Liljeström, M., A. Schiavini, and J. C. Reboreda. 2009. Chilean swallows (Tachycineta meyeni) adjust the number of feathers added to the nest with time of breeding. Wilson J. Ornithol. 121: 783-788.
36. Liljeström, M., A. Schiavini, and J.C. Reboreda. 2012. Time of breeding and female condition affect chick-growth in the Chilean swallow (Tachycineta meyeni). Emu 112: 157-161.
37. Massoni, V., F. Bulit, J. C. Reboreda. 2007. Breeding biology of the white-rumped swallow Tachycineta leucorrhoa in Buenos Aires Province, Argentina. Ibis 149: 10-17.
38. Brown, C. R. 1983. Vocalizations and behavior of violet-green swallows in the Chiricahua Mountains, Arizona. Wilson Bull. 95: 142-145.
39. Stager, M., E. Lopresti, F. A. Pratolugo, D. R. Ardia, D. Caceres, C. B. Cooper, E. E. Iñigo-Elia, J. Molina, N. Taylor, and D.W. Winkler. 2012. Reproductive biology of a narrowly endemic Tachycineta swallow in dry, seasonal forest in coastal Peru. Ornithol. Neotrop.l 23: 95-112.
40. Robbins, M. B., G. H. Rosenberg, F. S. Molina, M. A. Jacome. 1997. Taxonomy and nest description of the Tumbes swallow (Tachycineta [albilinea] stolzmanni). Ornithol. Monog. 48: 609-610.
